# EST analysis in *Ginkgo biloba*: an assessment of conserved developmental regulators and gymnosperm specific genes

**DOI:** 10.1186/1471-2164-6-143

**Published:** 2005-10-15

**Authors:** Eric D Brenner, Manpreet S Katari, Dennis W Stevenson, Stephen A Rudd, Andrew W Douglas, Walter N Moss, Richard W Twigg, Suzan J Runko, Giulia M Stellari, WR McCombie, Gloria M Coruzzi

**Affiliations:** 1 The New York Botanical Garden, 200th St. and Kazimiroff, Bronx, NY 10458-5126, USA; 2 New York University, Department of Biology 1009 Main Building, New York, NY 10003, USA; 3 Centre for Biotechnology, Tykistökatu 6, FIN-20521 Turku, Finland; 4 Genome Research Center, Cold Spring Harbor Laboratory, 500 Sunnyside Blvd, Woodbury, NY 11797, USA; 5 Biology Department, Duke University, Box 91000, Durham, North Carolina, 27708; 6 Department of Plant Biology, Cornell University, Ithaca NY 14850, USA

## Abstract

**Background:**

*Ginkgo biloba *L. is the only surviving member of one of the oldest living seed plant groups with medicinal, spiritual and horticultural importance worldwide. As an evolutionary relic, it displays many characters found in the early, extinct seed plants and extant cycads. To establish a molecular base to understand the evolution of seeds and pollen, we created a cDNA library and EST dataset from the reproductive structures of male (microsporangiate), female (megasporangiate), and vegetative organs (leaves) of *Ginkgo biloba*.

**Results:**

RNA from newly emerged male and female reproductive organs and immature leaves was used to create three distinct cDNA libraries from which 6,434 ESTs were generated. These 6,434 ESTs from *Ginkgo biloba *were clustered into 3,830 unigenes. A comparison of our *Ginkgo *unigene set against the fully annotated genomes of rice and *Arabidopsis*, and all available ESTs in Genbank revealed that 256 *Ginkgo *unigenes match only genes among the gymnosperms and non-seed plants – many with multiple matches to genes in non-angiosperm plants. Conversely, another group of unigenes in *Gingko *had highly significant homology to transcription factors in angiosperms involved in development, including MADS box genes as well as post-transcriptional regulators. Several of the conserved developmental genes found in *Ginkgo *had top BLAST homology to cycad genes. We also note here the presence of ESTs in *G. biloba *similar to genes that to date have only been found in gymnosperms and an additional 22 *Ginkgo *genes common only to genes from cycads.

**Conclusion:**

Our analysis of an EST dataset from *G. biloba *revealed genes potentially unique to gymnosperms. Many of these genes showed homology to fully sequenced clones from our cycad EST dataset found in common only with gymnosperms. Other *Ginkgo *ESTs are similar to developmental regulators in higher plants. This work sets the stage for future studies on *Ginkgo *to better understand seed and pollen evolution, and to resolve the ambiguous phylogenetic relationship of *G. biloba *among the gymnosperms.

## Background

**Figure 1:**
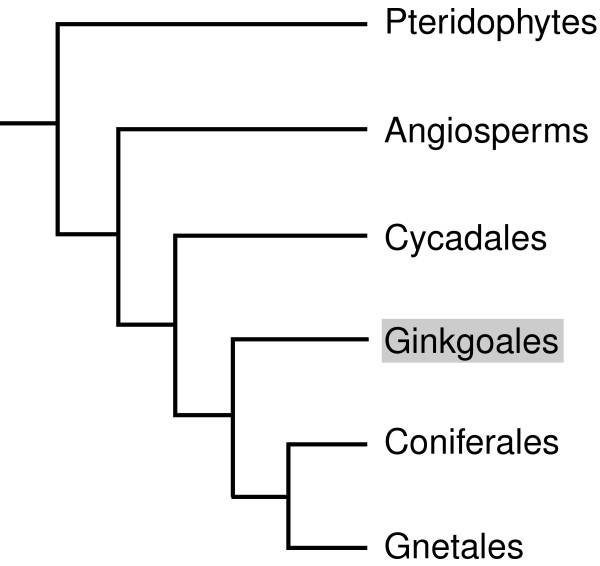
Gene tree of extant gymnosperms. *Ginkgo *displays characters suggesting it forms a basal subgroup among the gymnosperms with cycads. Alternately *Ginkgo *is a sister group of the conifers. Here the placement of *Ginkgo *is shown as ambivalent between these two scenarios.

*Ginkgo biloba *is a widely popular tree that is native to China and has been cultivated for well over a millennium. In Asia, *G. biloba *is used medicinally and its seeds are also a popular cuisine item. In the West, *Ginkgo *leaf extracts are commonly used for a variety of folk remedies (for review see: [1]) including as a treatment for improving cognitive function [2,3]. Today's *Ginkgo biloba *is the sole surviving species of an ancient group (Ginkgophytes) of seed plants that may even date from the Permian (approximately 150–200 million years ago) [4]. The genus *Ginkgo *itself goes back to the Jurassic period – approximately 170 million years ago [5]. Although it is widely believed that the survival of *G. biloba *depended upon Buddhist monks, who venerated the tree cultivated in their temple grounds, molecular evidence suggests that some stands in China (Wuchuan, Guizhou) are of natural origin representing vestige populations [6]. As a living fossil, *Ginkgo biloba *has changed little in morphology from its extinct relatives [5]. Along with the Cycadales, Coniferales and Gnetales, the Ginkgoales is one of four orders of non-flowering seed plants (gymnosperms) that form a sister group to the angiosperms (Figure 1).

Morphological [7,8] and molecular analysis have not yet succeeded in defining the precise phylogenetic hierarchy of the four gymnosperm clades [9]. Ginkgo potentially forms a sister group with the Coniferales (partly due to similar characteristics such as axillary branching and simple leaves). Another model, based on molecular sequence data, places *Ginkgo *with the Cycadales [10-12]. Interestingly cycads and *Ginkgo *both share certain plesiomorphic (ancestral) characters found in early fossil seed plants such as haustorial pollen [13,14], which release motile male gametes [15] as well as a large four celled opening in the neck of the archegonia [13,16]. Despite the presence of these and other early seed-plant characteristics, surprisingly little work has been performed on *Ginkgo *and cycads. Some recent molecular [17] and genomic [18] research on cycads have been conducted and molecular studies of *Ginkgo *genes have been initiated as well [19-21]. However, no genomic work on *Ginkgo biloba *has been completed to date.

**Figure 2:**
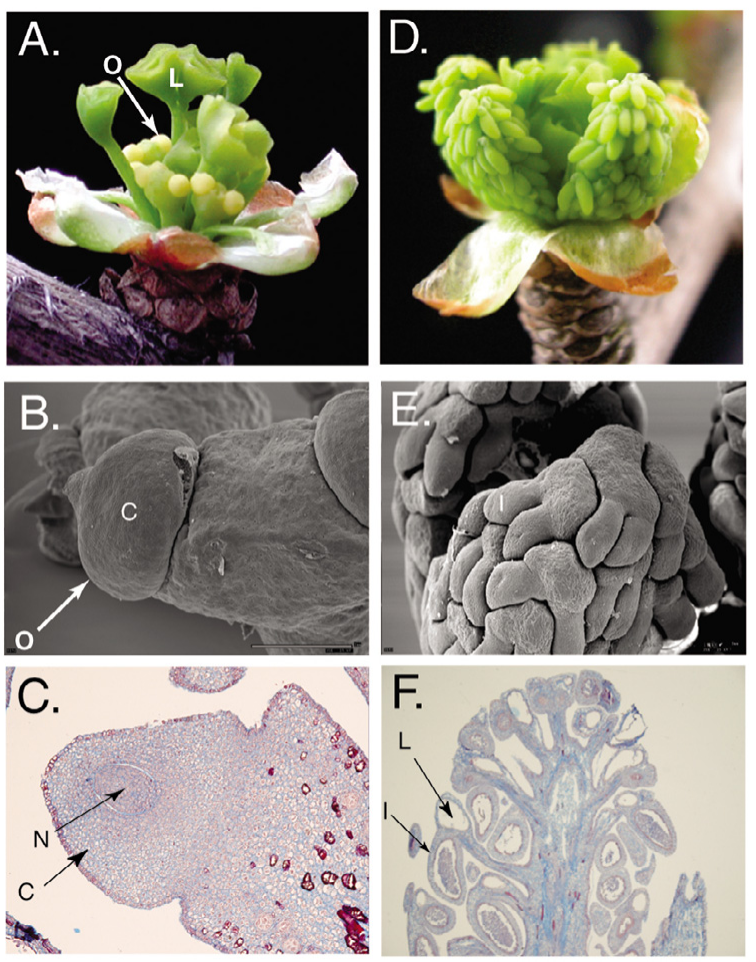
Ginkgo male and female short shoots. (A) The fertile female structure (megasporangia) has just emerged from the bud. Two ovules set on a green stalk are visible. Young, unfurled leaves, which have also emerged have extended above the megasporangia. The bracts, which originally enclosed the bud, are now completely opened below the leaves and megasporangia (B) Scanning EM of an ovule, which is completely enclosed by an integument. (C) A longitudinal cross section of the megasporangia reveals the integument enclosing the nucellus. (D) The male reproductive structure is a cluster of microsporangia. In the center of the bud are partly emerged leaves (E) Scanning EM shows two microsporangial lobes containing ripening pollen sacks attached to a stalk. (F) Longitudinal cross section shows a large mucilage containing cavity juxtaposed from the microsporangia filled with immature pollen. C integument; N, nucellus; I, microsporngia; L, mucilage cavity. O, ovule

To begin our genomic treatment of *Ginkgo biloba*, we focused our initial efforts on developing reproductive and vegetative tissues (Figure 2). Separating *Ginkgo *male and female structures at an early stage is straightforward because *Ginkgo *is strictly a dioecious plant (male and female organs on separate individuals). Organ emergence can generally be pinpointed to a specific time of the year in that both reproductive and vegetative tissues are regularly produced in the beginning of May at our collection site in New York. The reproductive structures, megasporangia bearing ovules (Figure 2A–C) (from female trees) and microsporangia bearing pollen (Figure 2D–F) (from male trees), emerge at the apex of short, determinate (spur) shoots. A discreet flush of leaves are also produced in male and female short-shoots (Figure 2A and 2D) [13,16,22]. Long shoots (not shown) exhibit indeterminate growth and yield only vegetative organs. Long-shoots are identifiable by their obvious longer internodes, whereas short shoots (Figure 2A and 2D) have telescoped internodes. Each season, short-shoots might exhibit extensive internode growth and be transformed into long-shoots and vice versa. Consequently, reproductive shoots can become vegetative or vegetative shoots can become reproductive.

Until now, little is known regarding the genetic regulation of development in the oldest living seed plants. In order to uncover the genetic controls directing growth and development in *Ginkgo biloba*, we generated expressed sequence tags (ESTs) from cDNA libraries of very young, recently emerged organs of fertile short-shoots where a large number of regulatory genes are expected to be present. Below is an analysis of these ESTs from *Ginkgo biloba. *In all three tissues examined, vegetative, microsporangia (male), and megasporangia (female), we found a large number of ESTs with similarity to angiosperm developmental genes. Conversely, a certain number of *Ginkgo biloba *ESTs were uncovered with homology to genes only found in gymnosperms and non-seed plants, including a set of *Ginkgo *ESTs that were only common to our cycad EST dataset, further strengthening their classification as gymnosperm specific.

## Results and discussion

### Construction of a cDNA library from *Ginkgo biloba *fertile and vegetative tissue

Young organs (Figure 2A and 2D) were collected during the spring from the opening buds of short shoots immediately after their emergence. At this stage, the megasporangium consists of an axis typically bearing two ovules (Figure 2A–C). The ovule is composed of a single integument surrounding a developing nucellus (Figure 2C). The male structure consists of a main axis bearing two or more microsporangia (Figure 2D–F). RNA was extracted from the following organs: megagasporangia, microsporangia and two sets of leaves collected from either male or female trees. mRNA isolated from all four tissues was used to construct four separate cDNA libraries. (Both male and female leaf sequences were pooled during subsequent bioinformatic analysis). Size fractionation was used to enrich for full-length cDNAs during library construction. From this cDNA library, 6,434 sequence reads (Expressed Sequence Tags, ESTs) were generated. All *Ginkgo biloba *EST reads have been deposited in GenBank. It was determined that 3,739 (58%) of the cDNA clones were over 500 bp long. 3618 of the reads were generated from the 5' end of the cDNA, and 2816 were sequenced from the 3' end. Cluster analysis on the EST sequence produced a unigene set of 3,830 contigs consisting of 2,851 singletons and 979 assemblies. Of the clusteredESTs, the longest contig was 2,172 bp. The entire unigene set or complete *Ginkgo *BLAST files can be downloaded at the following website [23]. Each *G. biloba *contig is given a numeric identifier. The constituent ESTs for each contig can be obtained at this website. Additional bioinformatic analysis of the *Ginkgo biloba *dataset can be accessed at the open Sputnik Comparative Genomics Platform at [24]. This site features sequence annotations, peptide sequence predictions, protein domain architectures and putative molecular markers (ISSRs) for the ginkgo EST derived unigenes. The sequence can be downloaded either as a fasta file, a clustered fasta file or as the derived peptide fasta file. In addition, BLAST analysis can be performed with the clustered ESTs from a given ginkgo organ against all genes in *Arabidopsis thaliana *or distinct plant clustered EST datasets using the ViCoGenTa program available at the New York Plant Genomics Consortium website [23].

### Ginkgo contig matches to genes in angiosperms, gymnosperms and non-seed plants

TBLASTX (expect < 1eX10-5) was used to compare the *G. biloba *unigenes against all available plant ESTs from TIGR (The Institute for Genomic Research) [25], and the Plant Genome DataBase (Plant GDB) [26]. ESTs from these databases were downloaded and clustered into unigenes, which were used in the comparison. Next the *Ginkgo *unigene set was compared against the *Arabidopsis *and rice genome annotated protein sequences downloaded from TIGR. All genes used in this comparison against *Ginkgo *were divided into one of three taxonomically relevant categories: 1. angiosperms, 2. gymnosperms, and 3. non-seed plants. The angiosperm category encompasses all annotated rice and *Arabidopsis *genes identified from their respective genomic sequences, as well as all higher plant ESTs. The majority of the gymnosperm ESTs came from the conifer groups pine and spruce but also include ESTs generated from the Plant Genomics Consortium containing ESTs from the two other gymnosperm clades, Cycadales and Gnetales. The non-seed plant category consisted of genes from all remaining plant ESTs including ferns, fern allies, bryophytes and algae available with the majority of the sequences originating from *Physcomitrella patens *and *Chlamydamonas reinhardtii*.

**Figure 3:**
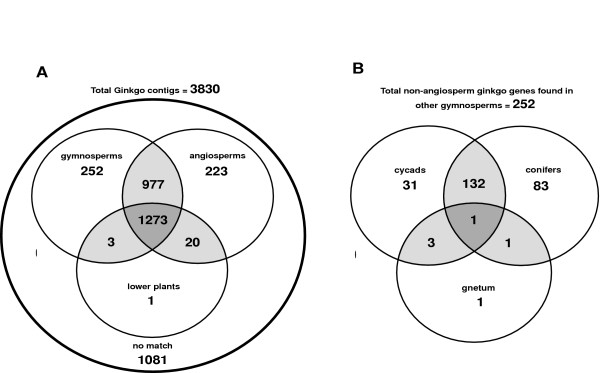
A Venn diagram illustrating the number of *Ginkgo *contigs with shared homology to genes found in non-seed plants, gymnosperms and/or angiosperms (A). A BLASTX E value >e 10-5 was used as a cut-off. (B) The *Ginkgo *contigs with similarity to gymnosperms but (no match to angiosperm genes) were further subgrouped according to their BLAST score homology (E value >e 10-5) within gymnosperm taxa.

A Venn diagram shown in Figure 3A displays the number of *Ginkgo *contigs, which are shared between one or more of the plant EST datasets at low BLAST stringency value (expect < 1eX10-5). From the Venn diagram, it can be seen that a majority of *Ginkgo *unigenes (2749/3830) match genes in other plants, and 1081 have no match to other plant genes. Of those 2749 *Ginkgo biloba *unigenes with matches to other plant genes, a subgroup of 256 unigenes had no corresponding match to genes in the angiosperm dataset. Of these 256 *Ginkgo *genes that do not match angiosperms, 4 also match genes in non-seed plants.

The 252 *Ginkgo biloba *unigenes that only match gymnosperm genes were next partitioned into matches between the three other gymnosperm orders: Cycadales, Coniferales, and Gnetales (Figure 3B). Since there are significantly more conifer unigenes (>60,000) than cycad unigenes (5459) that were used in this comparison, one would expect that the number of matches between *Ginkgo *and conifers would be significantly greater than matches between *Ginkgo *and cycads. However the actual number of matches between *Ginkgo *and conifers (215) is only slightly more then for *Ginkgo *and cycads (163). In other words despite the fact that there is over 10 times the number of conifer genes then cycad genes used in this comparison, *Ginkgo *matches to conifers are only 1.3 times greater then matches between *Ginkgo *and cycads. Of the matches between *Ginkgo *and gymnosperms, 31 match only cycads, (22 match *Cycas rumphii*, 6 match only unigenes from the cycad species, *Zamia furfuracea*, (753 contigs deposited in Genbank from Brenner et al, unpublished data); and 3 *Ginkgo *contigs match unigenes from both *Cycas rumphii *and *Zamia furfuracea*).

As one might expect, unigenes with matches to other plants are somewhat longer then those that have no match to other plants. Of all Ginkgo unigenes with matches to other plants, 89% are greater than 300 bp, whereas 72% are greater than 300 bp then those *Ginkgo *unigenes with no matches to other plants.

### Common genes between cycads and *Ginkgo*

Our comparative analysis of the *Ginkgo *EST dataset builds upon our results from a previous genomic study on the cycad, *Cycas rumphii *[18]. In our current analysis, three (CB090673:GinkgoA3816, CycadCB089620:GingkoA3730, and CycadCB089926:GinkgoA1532) of the fourteen unigenes from cycads previously found only among gymnosperms (after the full-length clone was sequenced), were also homologues to *Ginkgo *genes found only in gymnosperms. Considering the relatively small number of unigenes from *Ginkgo *(3,830) and cycads (4706) available for our comparative studies, the detection of the same gene match in *Ginkgo *and cycads with homology to only gymnosperms strengthens the argument that these genes are gymnosperms specific.

### *Ginkgo *matches to non-seed plants but not angisosperms

An additional four *Ginkgo *unigenes that are not found in angiosperms were detected in non-seed plants. Three of these *Ginkgo *unigenes (GinkgoA2411, GinkgoA3214 and GinkgoA325) match non-seed plants and other gymnosperm genes whereas the forth *Ginkgo *gene (GinkgoA2273) only matches non-seed plants with similarity to a gene in *Chlamydomonas*.

### Classification of *G. biloba *ESTs by functional categories

Each contig from the *Ginkgo *dataset was automatically assigned to a functional category (FunCat) based on its top match against the MIPS FunCat list of functionally annotated gene sequences from *S. cerevisiae *and *A. thaliana *databases using BLASTP. A non-stringent expect value (E-value) of < e-10 was chosen as the threshold. Table 1 below illustrates the relative fraction that each functional category comprises within the entire unigene set compared to our previous study in *Cycas rumphii *[18]. The four largest categories of *Ginkgo *ESTs according to this functional categorization are: "cellular organization" (19%), "metabolism" (11%), "unclassified proteins" (14%), and "protein synthesis" (8%). In general, these same categories are also the highest in the cycad EST library from our previous work, except for "protein synthesis", which appears increased in *Ginkgo*, whereas interestingly, the category of cell growth, cell division and DNA synthesis is reduced in *Ginkgo *compared to cycads.

**Table 1:** Placement of *Ginkgo *unigenes into functional categories (funcats). *Ginkgo *genes with a BLASTP expect value (E-value) of > e-10 were assigned into funcats based on their similarity score. The analysis was performed at the Centre for Biotechnology, Turku, Finland. Previous funcat analysis data from *Cycas rumphii *is shown.

Functional Category	Ginkgo%	Cycad%
Metabolism	11.0	10.3
Energy	5.0	5.1
Cell Growth, Cell Division and DNA Synthesis	5.4	9.2
Transcription	3.6	5.0
Protein Synthesis	8.4	5.8
Protein Destination	5.8	8.3
Transport Facilitation	2.0	1.3
Intracellular Transport	3.4	4.3
Cellular Biogenesis	3.5	3.3
Cellular Communication/Signal Transduction	3.5	4.2
Cell Rescue, Defense, Cell Death and Ageing	7.2	6.8
Ionic Homeostasis	0.5	0.1
Cellular Organization	18.8	21.6
Classification Not Yet Clear-Cut	7.3	4.6
Unclassified Proteins	14.1	10.3

### *Ginkgo *genes involved in development

Analysis of the *Ginkgo biloba *dataset revealed a number of ESTs with highest BLAST similarity to genes with known roles in higher plant developmental processes. A sampling of some of these genes is shown below (Table 2). These genes included the *Polycomb *gene *CURLY LEAF *[27,28] as well as *LATERAL ORGAN BOUNDARIES (LOB) *[29], *EARLY FLOWERING 5 (ELF5) *[30], *FLOWERING LOCUS T (FT) *[31], and *CONSTANS *(for review see [32] as well as five ESTs that match *MADS *box genes, some of which appear identical to previously cloned fragments of *MADS *genes from *Ginkgo biloba *including the *G. biloba *ortholog of *AGAMOUS *[19]. Other genes in our EST library have homologies to proteins that regulate development through protein turnover including *SPA-1 *[33]*COP1 *[34] and *COP9 *[35,36]. This sampling reveals that the EST dataset of *Ginkgo biloba *is a rich source of genes encoding proteins with known roles in development at the transcriptional as well as post-transcriptional stage.

**Table 2:** Similarity match of *Ginkgo *unigenes to genes involved in development. The *G. biloba *unigene set was compared to Genbank using a BLASTP cut-off score < e-5. The top match is listed under subject description. The organ(s) from which the listed ESTs were detected are: G = megagametophyte, I = microgametophyte, and L = leaf.

**Genes in *Ginkgo biloba *with similarity to developmental genes in other plants**			
Contig id.	organ	BLAST homology match	E-value

A2725	G	gi|1903019|emb|CAA71599.1| curly leaf [*A. thaliana*]	4.00E-37
A3095	I	gi|15231388|ref|NP_188001.1| LOB domain family protein [*A. thaliana*]	2.00E-21
A2815	I	gi|42541156|gb|AAS19471.1| EARLY FLOWERING 5 [*A. thaliana*]	1.00E-33
A3351	I	gi|4903139|dbj|BAA77836.1| extensive homology to FT (FLOWERING LOCUS T, AB027504)	4.00E-10
A241	I, L	gi|41323976|gb|AAS00054.1| CONSTANS-like protein CO1 [*Populus deltoides*]	1.00E-30
A1591	L	gb|AAG43405.1|AF172931_1 homeobox 1 [*Picea abies*]	5.00E-23
A1591	G	gi|14715183|emb|CAC44080.1| putative MADS-domain transcription factor DEFH7 [*A. majus*]	2.00E-39
A2737	G	gi|30230270|gb|AAM76208.1| AGAMOUS-like MADS-box transcription factor [*Ginkgo biloba*]	5.00E-28
A2850	I	gi|25307918|pir||S51935 probable MADS-box protein dal1 – Norway spruce dal1 [*Picea abies*]	5.00E-72
A629	G, L	gi|7446554|pir||T10751 MADS-box protein MADS9 – Monterey pine	6.00E-17
A352	G, I	gi|7446559|pir||T09571 MADS box protein MADS2 – Monterey pine	4.00E-75
A914	G, I	gi|18401293|ref|NP_565632.1| COP9 / CSN signalosome complex subunit [*A. thaliana*]	1.00E-45
A2730	G	gi|15225760|ref|NP_180854.1| COP1 regulatory protein [*A. thaliana*]	2.00E-60
A944	G, I	gi|30694320|ref|NP_849784.1|argonaute protein(AG01) [*A. thaliana*]	1.00E-56

## Conclusion

### The importance of *Ginkgo *for the study of plant evolution

As the sole remaining species of an ancient genus of plants which has survived nearly 170 million years from the Jurassic [5], *Ginkgo biloba *is a taxonomic and geographic relict that may be even older because fossils displaying a "ginkgophyte" vegetative morphology have been found as early as the Permian [4]. *Ginkgo *has a number of plesiomorphic (unspecialized) as well as apomorphic (derived) traits that make it a valuable tool to study the evolution of seed plants. Here we used a genomic approach to investigate the genes involved in regulating development in *Ginkgo *by creating an EST library from both reproductive and vegetative tissues.

Similar to our previous analysis in *Cycas rumphii*, our *Ginkgo *EST study has found significant BLAST homology between *Ginkgo *ESTs with plant genes in gymnosperms and non-seed plants but not in angiosperms. Since ESTs, even when clustered in contiguous genes, may not represent the complete gene [37], often one will find homology to angiosperm genes when the remaining *Ginkgo *gene sequence is revealed. For example in the gymnosperm, *Pinus taeda *EST collection, contigs of increasing length have a higher likelihood then shorter contigs matching a known gene in the *Arabidopsis *genome [38]. However, in this same study a significant subcategory of very long contigs (>1900 bp) have no homology to *Arabidopsis *[38]. It is likely that at least some of these long contigs with no match to angiosperm genes represent full length genes that are specific to the gymnosperm and/or seed-less plant clades. Our strategy to address this question involves screening for these same genes in additional taxa of gymnosperms, in the case of this study, *Ginkgo biloba. *In our analysis three *Ginkgo *genes that were only found in gymnosperms also matched the fourteen ESTs from our previous study of gymnosperm common cycad genes.

Along these same lines, our results suggest the presence of genes common to non-seed plants and gymnosperms that are not present in angiosperms. This non-seed plant/gymnosperm grouping is not surprising considering the fact that gymnosperms have morphologically common characters that are not found in the angiosperms – particularly in their reproductive structures. For example, the megagametophyte is highly reduced both in cell number and in structural organization in angiosperms when compared to gymnosperms. Although these results cannot say for certain that these genes are specific to non-seed plants and gymnosperms, or more specifically that these genes are found in gymnosperm structures that are not found in seed plant, it nonetheless represents an important starting point to correlate the presence or absence of gymnosperm genes in angiosperms and/or lower plants.

### Are cycads and *Ginkgo *sister taxa?

One result from our study found that the number of *Ginkgo *contig matches to conifers are only 1.3 times greater then matches between *Ginkgo *and cycads despite the fact that there is over 10 times the number of conifer genes then cycad genes used in this comparison,. Taken together these results might indicate a closer evolutionary association between *Ginkgo *and cycads then between *Ginkgo *and conifers. This bias towards cycad/*Ginkgo *similarity correlates with the fact that the majority of molecular phylogenetic studies place as the cycads sister group to *Ginkgo*. Hopefully, this preliminary data will encourage further phylogenomic studies to fully resolve the hierarchy among extant gymnosperm orders. Until the full genome sequence becomes available for key gymnosperm taxa, EST sampling provides an important initial step for large scale identification of molecular markers to generate robust phylogenetic trees.

### Developmental regulators in *Ginkgo*

In *Ginkgo biloba *we note here a variety of genes with similarity to developmental regulators in angiosperms. We also note below that homologues to some of these developmental regulators are also present in our *Cycas rumphii *library as either orthologs to those found in higher plants or at least, belonging to the same gene family. An EST from *Ginkgo biloba *that was detected in the megagametophyte library has high similarity to the *Arabidopsis CURLY LEAF *(*CLC) *gene, which belongs to the *Polycomb-*group proteins (PcGs). PcGs epigenetically regulate downstream target genes [28]. PcGs modify chromatin-protein complexes that repress homeotic gene transcription and influence cell proliferation. In *Arabidopsis *PcG genes have been shown to regulate MADS box genes [39]. The *CLC *protein product regulates the expression of *AGAMOUS *[27], a gene controlling floral organ identity [40]. Interestingly, an ortholog for angiosperm *AGAMOUS *was also detected in the *Ginkgo *megagametophyte library (Table 2). *Ginkgo AGAMOUS*, (previously named *GBM5*) was identified in a study where the MADS domains were examined in *Ginkgo *[19]. In this work *Ginkgo AGAMOUS *was shown via RT-PCR to be expressed in not only female but also in male and vegetative tissue. In our analysis, five total MADS box homologues were also detected in the *Ginkgo *EST dataset. Three of the *Ginkgo *ESTs from our library, GinkgoA2340, GinkgoA2730 and GinkgoA2850, are perfectly identical to the MADS domain gene fragments previously cloned by [19] as degenerate PCR products. The other two unigenes from our dataset have homologies to MADS genes (GinkgoA629 and GinkgoA352), but do not specifically match any of the PCR fragments isolated in their study. These two MADS box unigenes either do not include the small region amplified in their degenerate PCR screen or could alternative be unique MADS genes not isolated in their study. Unlike the degenerate primer approach used to isolate MADS genes, our EST approach offers the additional advantage of cloning entire genes or at least substantially large gene fragments. Among the few developmental genes examined in gymnosperms, most attention has focused on the expression of MADS homologs [41,42].

Other developmental genes found in the *Ginkgo *EST library include those with homology to regulators of flowering such as *EARLY FLOWERING 5 (ELF5)*, which controls the levels of the gene *FLC*, which itself is a central regulator of flowering [30]. Another *Ginkgo *EST includes *FLOWERING LOCUS T (FT)*, which belongs to a small family of genes (*FT/TFL1) *that act to promote flowering as a downstream component from *CONSTANS *[43]. *CONSTANS *is a transcription factor that has a critical role integrating circadian rhythms and light signals (for review see [32]). As one would expect an EST homolog for the *CONSTANS *gene family was found in *Ginkgo*. *CONSTANS *belongs to a large gene family, which may have redundant roles in plants [44]. Not surprisingly, we also found homologs to *CONSTANS *in our previous study on cycad leaf ESTs [18]. In that flowering plants are believed to have evolved from gymnosperms, a survey of *CONSTANS*, *ELF*, and *FT *in gymnosperms, particularly in very young reproductive tissue might help define the origins of reproductive induction in non-flowering plants. Among the other genes related to developmental regulators includes a homologue to *LATEROL ORGAN BOUNDARIES (LOB) *domain gene family which in *Arabidopsis *has over 40 members [29]. The molecular mechanism of *LOB *domain containing genes is unknown, but one gene in *Arabidopsis, ASYMMETRIC LEAVES2*, is required for normal leaf development, by potentially acting as a regulatory repressor of *KNOX *genes [45]. A *KNOX *homolog is also present in our EST library and was found in male reproductive tissues and *HOX *genes were also detected in our previous analysis in *C. rumphii*

Another important component regulating development occurs at the level of protein degradation. A gene recognized in our EST library includes *COP1. *COP1, serves as an E3 ubiquitin targeting photomorphogenic factors such as *HY5 *for degradation [33]. Another *Ginkgo *EST from the library has highest similarity to *COP9*. In our previous EST analysis in *Cycas rumphii *an EST was also isolated with similarity to COP9 [18]. COP9 is a subunit of the COP9 signalosome complex that controls multiple signaling pathways that regulate development in all eukaryotes [35,36]. In *Arabidopsis*, the *cop9 *and *cop1 *mutants are constitutively photomorphogenic in dark grown seedlings [46]. Unlike angiosperms, seedlings from conifers are constitutively photomorphogenic when grown in the dark [47,48]. In *Ginkgo*, chlorophyll and chloroplast development is completely dependent on light, however this process proceeds at a markedly slower pace then in flowering plants. That is, photomorphogenic development in *Ginkgo *seedlings is strongly delayed after transfer from dark grown conditions to light grown conditions when compared to seed plants [20,21]. The dark grown phenotype of cycads is unreported. Considering this variability in photomorphic development among and between the gymnosperms and the angiosperms, the discovery of genes encoding photomorphogenic regulators in gymnosperms will help understand the evolution of photomorphogenesis in seed plants.

Taken together, our genomics analysis of *Ginkgo biloba *is an important additional step to analyze the role of molecular development of early seed plants. Thus the stage is set to further determine the role of these genes during the development of ancillary structures found between *Ginkgo*, cycads and other gymnosperms with higher plants as well as the role of those in structures that are unique to gymnosperms and/or the non-seed plants as a step to understand the evolution of the seed plant habit.

## Methods

### Tissue collection and library construction and DNA purification

Newly emerged microsporangia from accession 76163B, megasporangia from accession 76163D, and immature leaves from both accessions were collected from newly opened buds of *Ginkgo biloba *growing in the New York Botanical Garden outdoor collection on April 12, 2002. Organs were snap frozen in liquid nitrogen. RNA was collected from each organ and a cDNA library was constructed from fractionated cDNA according to [49].

### Microscopy

Ginkgo apices were collected on April 19. Bract tissues were removed from the apex leaving the leaves and reproductive tissue, which was fixed in FAA (50% ethanol, 5% glacial acetic Acid, 3.7% formaldehyde) under vacuum (20 In. Hg) at room temperature. Fresh FAA was vacuum infiltrated two additional times. Tissue was stored in 70% ethanol at 4°C.

For histology, tissue was prepared by sequential (overnight 4 C incubation at each alcohol grade) dehydration in 80, 90, 95 and finally 100% ethanol plus Eosin Y (National Medicinal Products) followed by two treatments in 100% ethanol for 2 hours at room temperature. The tissue was next placed in a 1:1 solution of ethanol and toluene, then twice in toluene alone, each time for 2 hours at room temperature. The tissue was then placed in toluene with a quarter-volume of paraffin (PARAPLAST X-TRA^® ^(Fisher)) chips at 60°C overnight. The tissue was then embedded in melted paraffin with six wax changes over the course of three days at 55°C. Apices were sectioned on a MICROM HM 355 microtome. 8 μm thick sections were taken using a blade angle of 9°. The tissue was stained with Astra Blue and Safranin. After mounting on slides, sections were imaged using a Nikon DXM1200F digital microscope camera.

For scanning electron microscopy, fixed materials were dissected, dehydrated in ethanol and critical point dried in a Denton critical point dryer. Dried materials were affixed to aluminium E. M. stubs and coated with between 80–240 A of palladium in a Hummer II Sputter Coater. Coated materials were then observed using a Jeol scanning electron microscope at 15 or 20 kV. Images were digitally recorded and evaluated using Adobe Photoshop 9.0.

### EST sequencing and gene analysis

Plasmid DNA was collected as described in the manual (Stratagene), catalogue number 200450 in the in vivo mass excision section. Sequence analysis was performed at CSHL using an ABI 3700 Capillary sequencer for separation and nucleotide detection. Reactions were performed using a 1/16 Big Dye Terminator. Sequencing was performed with either the -21 M13 forward and/or reverse primer. ESTs were assembled using Phrap [50,51] and clustered into contigs using the CAP3 program [52]

### Peptide extraction

Peptide sequences were derived for all unigenes using the ESTScan application [53] run with the default parameters. Prior to the ESTScan predictions, a *Ginkgo *species-specific ESTScan model was created. ESTScan was trained with *Ginkgo *ORFs identified from the best match of BLASTX analyses performed on the unigene sequence against the Swissprot protein database. All BLASTX matches were filtered using the arbitrary expectation value of 1e-10.

### Sequence annotation

Sequence annotation on each of the *Ginkgo *cluster consensus sequences and derived peptides were performed within the openSputnik application [54]. Results were assessed for possible contamination by searching for homology to the *E. coli *and human genomes and were scored for homology to a wide range of non-coding RNAs and plant chloroplast and mitochondrial genomes. Homology searches were performed using the BLAST application [55] and results were filtered using the expectation value < 1e-10. Functional assignment was performed on both cluster consensus sequence and the peptide sequence. Assignments were made using BLASTX and BLASTP respectively against the MIPS catalogue of functionally assigned proteins (funcat) [50,51], tentative functional assignments were filtered using the expectation value < 1e-10.

### Categorization of *Ginkgo *contigs

All *Ginkgo *contigs sequences were aligned against a PlantEST database using TBLASTX [55] and BLASTX against the NR (aa) database. The PlantEST database was created by downloading all plant ESTs in GenBank and assembling them using Phrap [50,51]. Todd Wood from Clemson University provided the PERL script that creates the PlantEST databases as described above. The NR (aa) database is a non-redundant database of protein sequences from GenBank.

### Determination of gymnosperm specific genes

All available plant ESTs were downloaded from GenBank and separated into three datasets consisting of angiosperms (monocots and dicots), gymnosperms, or non-seed plants (ferns, mosses and algae). Downloaded ESTs were assembled using Phrap [50,51]. All matches with an expect value < 1e 10-5 are considered significant.

## Authors' contributions

EB conceived of this project. He participated in its design, experiments and drafted the manuscript. DS and GC also conceived this project and participated in its design and coordination. MK played the major role in the bioinformatics analysis. SAR performed the funcat analysis and built the Sputnik website, AD performed the scanning electron microscopy work, WM and GS performed the histological sectioning, RT and SJR performed the cDNA library construction, RM facilitated the EST sequencing. All authors read and approved the final manuscript.
